# The Effect of Coatings on the Affinity of Lanthanide Nanoparticles to MKN45 and HeLa Cancer Cells and Improvement in Photodynamic Therapy Efficiency

**DOI:** 10.3390/ijms160922415

**Published:** 2015-09-16

**Authors:** Takashi Sawamura, Tatsumi Tanaka, Hiroyuki Ishige, Masayuki Iizuka, Yasutoshi Murayama, Eigo Otsuji, Akihiro Ohkubo, Shun-Ichiro Ogura, Hideya Yuasa

**Affiliations:** 1 Department of Life Science, Graduate School of Bioscience and Biotechnology, Tokyo Institute of Technology, J2-10, 4259 Nagatsuta-cho, Midori-ku, Yokohama 226-8501, Japan; E-Mails: sawamura.t.ab@m.titech.ac.jp (T.S.); tatsumiya2015@gmail.com (T.T.); ishige.h.ab@m.titech.ac.jp (H.I.); masayuki.iizuka.12@gmail.com (M.I.); ohkubo.a.aa@m.titech.ac.jp (A.O.); sogura@bio.titech.ac.jp (S.-I.O.); 2 Division of Digestive Surgery, Department of Surgery, Kyoto Prefectural University of Medicine, 465 Kajii-cho, Kawaramachihirokoji, Kamigyo-ku, Kyoto 602-8566, Japan; E-Mails: murayama@koto.kpu-m.ac.jp (Y.M.); otsuji@koto.kpu-m.ac.jp (E.O.)

**Keywords:** lanthanide nanoparticle, ALA, near-infrared, up-conversion luminescence

## Abstract

An improvement in photodynamic therapy (PDT) efficiency against a human gastric cancer cell line (MKN45) with 5-aminolevulinic acid (ALA) and lanthanide nanoparticles (LNPs) is described. An endogenous photosensitizer, protoporphyrin IX, biosynthesized from ALA and selectively accumulated in cancer cells, is sensitizable by the visible lights emitted from up-conversion LNPs, which can be excited by a near-infrared light. Ten kinds of surface modifications were performed on LNPs, NaYF_4_(Sc/Yb/Er) and NaYF_4_(Yb/Tm), in an aim to distribute these irradiation light sources near cancer cells. Among these LNPs, only the amino-functionalized LNPs showed affinity to MKN45 and HeLa cancer cells. A PDT assay with MKN45 demonstrated that amino-modified NaYF_4_(Sc/Yb/Er) gave rise to a dramatically enhanced PDT effect, reaching almost perfect lethality, whereas NaYF_4_(Yb/Tm)-based systems caused little improvement in PDT efficiency. The improvement of PDT effect with the amino-modified NaYF_4_(Sc/Yb/Er) is promising for a practical PDT against deep cancer cells that are reachable only by near-infrared lights.

## 1. Introduction

**Figure 1:**
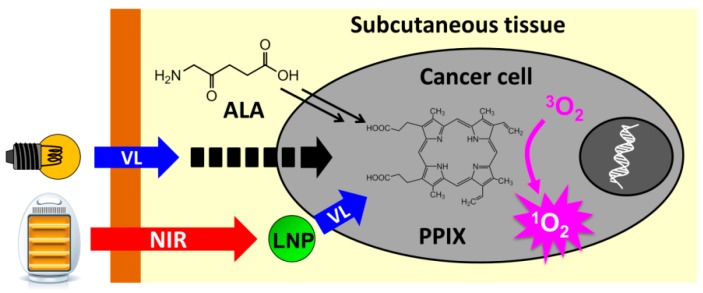
Photodynamic therapy with protoporphyrin IX (PPIX) as a photosensitizer. Uptake of 5-aminolevulinic acid (ALA) by a cancer cell leads to the accumulation of PPIX. A visible light (VL) may suffer from low penetrativity, resulting in failure to excite PPIX by epidermal irradiation. Lanthanide nanoparticles (LNP), if delivered near the cancer cell, will successfully excite PPIX by irradiation with high penetrative near-infrared (NIR) light.

Photodynamic therapy (PDT) of cancer is a potential substitute for chemotherapy and radiotherapy, because PDT enables a timely generation of cancer-killing molecules such as singlet oxygen (^1^O_2_) in a restricted area by a lighting manipulation, and the light irradiation is much safer for normal cells than radiation [1,2,3,4]. Most PDT systems employ a photosensitizer as the active oxygen generator, which is selectively incorporated in cancer cells and converts triplet oxygen (^3^O_2_) into ^1^O_2_ on light irradiation (Figure 1).

If a cancer patient is administered with 5-aminolevulinic acid (ALA) as a precursor of heme, photosensitizing protoporphyrin IX (PPIX) is selectively accumulated in cancer cells, owing to their impaired heme synthesis [5,6,7]. Blue light-sensitized PPIX can convert ^3^O_2_ into ^1^O_2_ in about 60% quantum yield [8]. The singlet oxygen is converted into several forms of active oxygen and these reactive molecules act as poisons to cancer cells [2]. Their limited lifetimes prevent the active oxygen molecules from diffusing out of cancer cells and damaging the surrounding normal cells [9]. A major advantage of ALA-PDT is the endogenous production of PPIX selectively in cancer cells, which requires no efforts to deliver a sensitizer into cancer cells, since ALA intake is promoted and PPIX consumption for the heme synthesis is suppressed in cancer cells [2].

**Figure 2:**
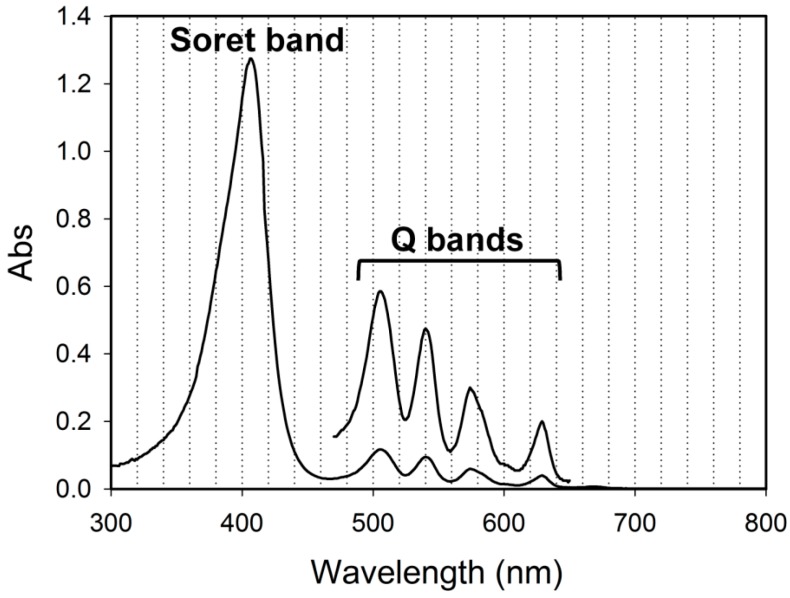
Absorption spectrum of PPIX. The five-fold expanded spectrum within Q bands region (470–650 nm) is overlaid for visual clarity.

A major drawback in common with all the PDT methods is the low skin penetrability of the excitation light [10,11]. PPIX has a strong Soret absorption band at 405 nm and four relatively strong Q bands between 500 and 630 nm, 630 nm being of longest in wavelength with about 30-fold smaller absorption coefficient than that of the blue light (Figure 2) [12]. The red 630 nm light is generally used for ALA-PDT, because its penetrativity through tissue is 10 times as high as the blue light penetration depth [10]. However, the best light penetrability (4–7-fold of the light at 630 nm) should be achieved with the wavelengths in so-called near-infrared (NIR) window (900–1100 nm) [10].

In order to cultivate an NIR-induced ALA-PDT for a human gastric cancer cell line (MKN45), we have exploited up-conversion lanthanide nanoparticles (LNP) [13], which emit the lights with wavelengths corresponding to the Soret band (405 nm) and a Q band (540 nm) of PPIX on excitation with NIR light (980 nm) [14,15]. Here we call this photosensitizing process as NIR-LNP-PPIX-PDT system. NIR laser irradiation at MKN45 co-incubated with ALA and LNP caused cell death [13]. Whereas the absorption of PPIX at 405 nm is about 15 times higher than that at 540 nm, LNP emitted about four-times less powerful light at 405 nm relative to the green light. Furthermore, LNP was only dispersed in the incubation medium and no efforts were made to recruit LNP near the cells so that the emitted lights efficiently reached the targets.

In this study, we explored an LNP-coating to effectively aggregate LNP on the surface MKN45 in order to improve the PDT efficiency. We also examined another LNP including Tm^3+^ as the emitter with strong emissions at 360 and 429 nm, which partly overlap the Soret band of PPIX.

## 2. Results and Discussion

### 2.1. Characterization of LNPs

**Figure 3:**
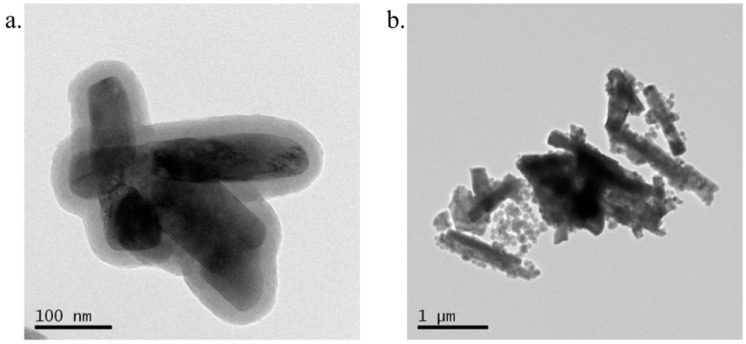
Transmission electron microscopy (TEM) images of (**a**) LNP(Er)AP; (**b**) LNP(Tm)AP. LNP: lanthanide nanoparticle; A1P: aminopropyl.

**Figure 4:**
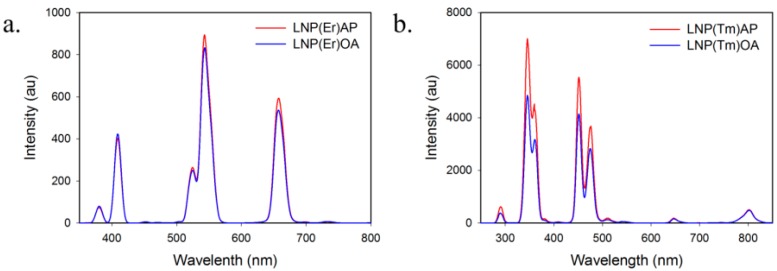
Up-conversion luminescence spectra (λ_ex_ 980 nm) of (**a**) LNP(Er)OA and LNP(Er)AP; (**b**) LNP(Tm)OA and LNP(Tm)AP. LNP: lanthanide nanoparticle; OA: oleic acid; AP: aminopropyl.

LNP(Er) and LNP(Tm) with compositions, NaYF_4_(Sc^3+^/Yb^3+^/Er^3+^, 30/18/2) and NaYF_4_(Yb^3+^/Tm^3+^, 20/0.7), were prepared as we previously reported [16] according to the methods of Liu’s group [17]. In these LNPs, Na^+^, Y^3+^, and F^−^ compose a host lattice, Yb^3+^ behaves as a sensitizer, and Er^3+^ and Tm^3+^ are emitters [18]. For LNP(Er), Sc^3+^ was selected as a co-dopant that is known to enhance the up-conversion emission strength [19]. To maximize the emission strength of LNP for an efficient PDT and focus on the affinity of LNPs with the cancer cells, we sacrificed homogeneity of LNP, because the mono-dispersed LNPs tended to have very weak emissions with more than three orders of magnitude in our hands. These LNP cores were coated with silica (Si) and an aminopropyl (AP) group according to the reported methods [20,21]. A ninhydrin test indicated an amino-group incorporation of 41 nmol/mg on LNP(Er). Transmission electron microscopy (TEM) images of aminopropyl-coated LNPs, LNP(Er)AP and LNP(Tm)AP, showed the silica coating layers as demonstrated by the semitransparent films formed around LNP cores with a thickness of about 20 nm (Figure 3 and Figure S1 for expanded images). Dynamic light scattering (DLS) measurements indicate that AP modification of OA-coated LNP has little effect on the hydrodynamic diameters (Figure S2): LNP(Tm)AP formed larger aggregations with an average size of 670 nm as compared with that of LNP(Er)AP (300 nm), which is consistent with the TEM images. LNP(Er)s showed relatively strong emissions at 409, 543, and 656 nm and LNP(Tm)s at 346, 360, 429, and 475 nm (Figure 4 and Figure S3). These emission strengths little changed after modifications with AP (Figure 4).

### 2.2. Affinity of the Surface Modified LNPs with a Cancer Cell

**Figure 5:**
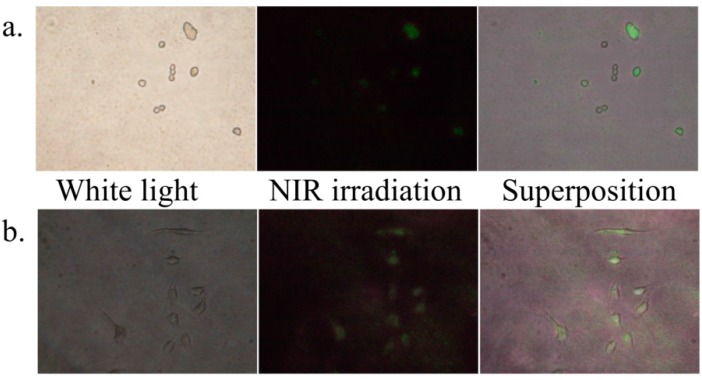
(**a**) MKN45 cells incubated with LNP(Er)AP under white light (**left**) and near-infrared light with 980 nm laser diode (**middle**) and the superposition of them (**right**); (**b**) HeLa cells incubated with LNP(Er)DAB under white light (**left**) and near-infrared light with 980 nm laser diode (**middle**) and the superposition of them (**right**). LNP: lanthanide nanoparticle; AP: aminopropyl; DAB: diaminobutane.

Compared with the other cancer cells, MKN45 cells lack the information about targeting strategies for drug delivery [22,23]. Since our primary interest in this study was the extent to which PDT effect is amplified by accumulating LNP near cancer cells from the interstitial fluid, we started from searching simple functional groups instead of antibodies or peptides. We prepared oleic acid (OA-), Si-, AP-, polyethylene glycol (PEG-), azelaic acid (AA-), chitosan (CHI-), polyethyleneimine (PEI-), diaminobutane (DAB-), folic acid (FOL-), and ALA-coated LNP(Er)s (preparation methods and quantifications are described in Supplementary Information). These surface-modified LNPs were incubated with MKN45 or HeLa cells. Among the assays for LNPs-cancer cell affinity, only the combinations of LNP(Er)AP/MKN45 and LNP(Er)DAB/HeLa showed obviously positive results: the green color of LNP emission on irradiation with 980 nm laser diode was superimposable on the cancer cells (Figure 5), suggesting that these LNP(Er)s have affinity to the cancer cells. The other combinations were negative or ambiguous (Figure S4). It is likely that the positive charge of LNP(Er)AP and LNP(Er)DAB interacted with the negative charge of the cell-membrane phospholipids and thus the affinities between these LNPs and the cancer cells were not caused by selective interactions. However, it should be noted that the amino functions in different platforms, AP and DAB, gave an apparently different selectivity toward MKN45 and HeLa, respectively. HeLa cells have been suggested to uptake positively charged nanoparticles mainly via macropinocytosis or a dynamin-dependent mechanism [24]. On the other hand, MKN45 has not been studied about the nanoparticle uptake mechanisms. The different uptake selectivity may be due to a difference in the uptake mechanisms between MKN45 and HeLa.

### 2.3. PDT Effects of Amino-Modified LNPs toward MKN45

**Figure 6:**
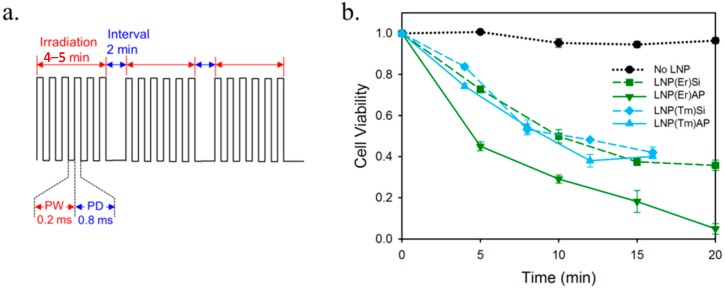
(**a**) The pulse modulation mode of NIR laser irradiation for the photodynamic therapy (PDT) assay: 4- or 5-min on and 2-min off with a pulse width (PW) of 0.2 ms and a pulse delay (PD) of 0.8 ms; (**b**) MKN45 cell viability by irradiation with a diode laser (976 nm, 62.1 W/cm^2^). The pulse irradiation for 5 min (Er) or 4 min (Tm) was repeated with 2 min intervals.

In the previous section, we found that AP-modified LNPs have an affinity with MKN45 cells. We thus studied the NIR-LNP-PPIX-PDT system on MKN45, using LNP(Er)Si, LNP(Er)AP, LNP(Tm)Si, and LNP(Tm)AP as the up-converters to activate PPIX-based ^1^O_2_ production (Figure 6). MKN45 was incubated with ALA and LNP for 24 h and then NIR irradiation was performed. NIR irradiation was carried out with a pulse laser, in which a manual on-off operation in the order of minutes was superimposed on the milliseconds laser pulse to alleviate a harmful temperature increase (Figure 6a). After a further incubation overnight, the cell viability was evaluated by assessing the cell’s capability of reducing 3-(4,5-dimethylthiazol-2-yl)-2,5-diphenyltetrazolium bromide into a purple-colored formazan (MTT assay) [25]. LNP(Er)Si, LNP(Tm)Si, and LNP(Tm)AP showed a similar PDT effect: half of the cancer cells were killed after the 10-min NIR pulse irradiation programs and cell lethality did not exceed 60% within 20 min (Figure 6b). The relatively strong emissions of LNP(Tm) at 346 and 429 nm are likely to be responsible for the excitation of Soret band of PPIX at 405 nm. However, the peak gaps between the LNP emissions and PPIX absorption, *i.e.*, Δ59 and Δ24 nm, might have been so large that their PDT effects did not exceed that of LNP(Er) with the emission peaks at 409 and 543 nm, which well overlap PPIX absorption of Soret and Q bands, respectively. Although the amino group of LNP(Tm)AP was expected to enhance PDT effect by its affinity to the cancer cells, the difference in PDT effects between LNP(Tm)Si and LNP(Tm)AP was marginal. On the other hand, a dramatic amino group effect was observed with LNP(Er)AP, in which a 20-min NIR pulse irradiation set caused almost 0% cell viability, while the same irradiation set with LNP(Er)Si left about 40% of the cancer cells alive. The enhancement of PDT effect is highly likely due to a more efficient excitation of PPIX from the cell-surface or intracellular LNP(Er)AP than that from intercellular LNP(Er)Si. The amino group effect stood out in the first five minutes: the cell lethality with LNP(Er)AP reached as much as 60%, as compared with 25% for LNP(Er)Si. It is suggested that close proximity of LNP, the irradiation source, prevented PPIX from the photo-bleaching before ^1^O_2_ production. In summary, LNP(Er)AP showed a most efficient PDT effect toward MKN45 cancer cells, while the other LNPs, LNP(Er)Si, LNP(Tm)AP, and LNP(Tm)Si, were evenly less effective.

## 3. Experimental Section

### 3.1. General

All reagents and materials were obtained from commercial suppliers and used as received. Rare earth chlorides YCl_3_·6H_2_O (99.99%), YbCl_3_·6H_2_O (99.99%), ErCl_3_·6H_2_O (99.99%), TmCl_3_·6H_2_O (99.99%), ScCl_3_·xH_2_O (99.99%) and all the coupling reagents were purchased from Sigma-Aldrich Co., Ltd. (St. Louis, MO, USA). Oleic acid (OA) was purchased from Kanto Chemical Co., Inc. (Tokyo, Japan). Potassium permanganate and sodium periodate were purchased from Kokusan Chemical Co., Ltd. (Osaka, Japan). The other inorganics and solvents were purchased from Wako Pure Chemical Industries Ltd. (Mie, Japan). Deionized (DI) water was used throughout the LNP preparation. MKN45 was purchased from Riken BRC Cell Bank (Tsukuba, Ibaraki, Japan). The preparation methods of surface-modified LNPs are described in Supplementary Information. The size and morphology of LNPs were determined by JEM-2010F field emission transmission electron microscopy (FE-TEM) (JEOL Ltd., Tokyo, Japan) at 200 kV. Dynamic light scattering (DLS) measurements were carried out on a Zetasizer nano series from Malvern Instruments Ltd. (Malvern, UK). Luminescence emission spectra were recorded on JASCO FP-8500 fluorospectrometer with an external laser diode excitation source (300 mW, 980 nm, THORLABS, Inc. (Shanghai, China)). NIR irradiation was performed with a laser diode setup of Asahi Data Systems, Ltd. (Kawasaki, Japan): FCSE01-4-976 laser diode and ALP-7603CB laser driver equipped with an FG-274 function generator (TEXIO Technology Co., Kanagawa, Japan). UV-visible absorbance was recorded with a USB2000+XRI-ES photospectrometer equipped with a DH-2000 light source (Ocean Optics Co., Dunedin, FL, USA).

### 3.2. Adhesibility of LNP to Cells

MKN45 cells were incubated (37 °C, 5% CO_2_) in an RPMI-1640 culture medium containing l-glutamine (584 mg/L), 10% (*v*/*v*) heat-inactivated fetal bovine serum, 100 U/mL penicillin, and 100 μg/mL streptomycin in a 10-cm dish. The medium was aspirated, the cells were washed with 2 mL of PBS buffer, then cell desquamation was performed with a trypsin solution (1 mL, 2.5 g/L) in PBS containing 1 mmol/L EDTA, 10% fetal bovine serum, and a small amount of phenol red as a pH indicator. After 5-min incubation (37 °C, 5% CO_2_), 3 ml of the RPMI-1640 culture medium was added to terminate the enzyme reaction. The living cell number in a 10 μL cell mixture was counted on a hemocytometer by trypan blue exclusion staining. The cell mixture (4 mL) was centrifuged for 5 min at 1100 rpm and the supernatant was replaced with 2 mL of the RPMI-1640 culture medium. The cell mixture was seeded in a 3-cm dish at a density of 1.0 × 10^6^ per well, and incubated overnight. The medium was aspirated, cells were washed with 100 μL PBS buffer, and a medium (100 μL) containing LNP (1 mg/mL) was added. After incubation for 24 h, the cells were observed with a phase-contrast microscope (Olympus CKX41) under white light or on irradiation with the NIR diode laser and pictured with a CCD camera (Olympus E-620).

### 3.3. In Vitro PDT Assay

MKN45 cells were incubated and desquamated as described above in the section 3.2. The cell mixture was seeded in a 96-well plate at a density of 2.0 × 10^4^ per well, and incubated overnight. The medium was aspirated, cells were washed with 100 μL PBS buffer, and a medium (100 μL) containing ALA (2 mmol/L) and LNP (1 mg/mL) was added. After incubation for 24 h, the cells in each well were irradiated with the NIR diode laser from 10 cm above the well plate. After overnight incubation, the medium was aspirated and cells were washed with 100 μL per well of PBS buffer and 100 μL of medium was added. Cell viability was quantitated with the MTT assay. The solution of 10% MTT in methanol was diluted 10 times with PBS and 10 μL of it was added to each well. After 4-h incubation, 100 μL/well of 10% SDS was added and incubated overnight. The absorbance of the mixture at 570 nm was measured on a microplate reader (Model 550, Bio-Rad Laboratories, Inc. (Berkeley, CA, USA)), in which absorbance at 650 nm was set to zero as the baseline.

## 4. Conclusions

OA-, Si-, AP-, PEG-, AA-, CHI-, PEI-, DAB-, FOL-, and ALA-coated LNP(Er)s and LNP(Tm)s were prepared. Among them, AP-coated LNP(Er) and DAB-coated LNP(Er) showed affinities to MKN45 and HeLa cells, respectively. The affinity of an LNP with cancer cells means that the irradiation light source is closer to PPIX than that diffused in intercellular space and thus NIR-LNP-PPIX-PDT is expected to be more efficient. We conducted NIR-LNP-PPIX-PDT assays with AP-coated LNP(Er), Si-coated LNP(Er), AP-coated LNP(Tm), and Si-coated LNP(Tm) to examine the effect of the affinity to cancer cells and the difference of the emitter dopants, Er and Tm. As a result, Si-coated LNP(Er), AP-coated LNP(Tm), and Si-coated LNP(Tm) showed similar PDT effects, about 60% lethality. On the other hand, AP-coated LNP(Er) indicated nearly a perfect lethality with almost 0% cell viability. The result is promising for further studies toward a practical PDT with high penetrative NIR irradiation.

## Supplementary Materials

Supplementary materials can be found at http://www.mdpi.com/1422-0067/16/09/22415/s1.
