# Supplementary material for: The Effect of Coatings on the Affinity of Lanthanide Nanoparticles to MKN45 and HeLa Cancer Cells and Improvement in Photodynamic Therapy Efficiency
Source: Int J Mol Sci. 2015 Sep 16;16(9):22415–24. doi: 10.3390/ijms160922415 (PMC4613315; doi:10.3390/ijms160922415)
Supplement: Supplementary file 1 [file ijms-16-22415-s001.pdf]

# Supplementary Information

## Abbreviations

AA: azelaic acid, ALA: 5-aminolevulunic acid, AP: aminopropyl, CHI: chitosan, DAB: 1,4-diaminobutane, DIPEA: diisopropylethylamine, DLS: dynamic light scattering, DMAP: 4-dimethylaminopyridine, DMF: *N*,*N*-dimethylformamide, DMSO: dimethyl sulfoxide, Fmoc: 9-fluorenylmethyloxycarbonyl, FOL: folic acid, HATU: 1-[*bis*(dimethylamino)methylene]-1H-1,2,3-triazolo[4,5-b]pyridinium 3-oxid hexafluorophosphate, LNP: lanthanide nanoparticle, OA: oleic acid, PEG: polyethylene glycol, PEI: polyethyleneimine, Si: silica, TEM: transmission electron microscopy.

*SI.1. Preparation of LNP(Er)Si*

To a stirred solution of LNP(Er)OA (60 mg) dispersed in cyclohexane (10 mL) was added poly(oxyethylene) nonylphenyl ether (IGEPAL® CO-520, (Sigma-Aldrich Co., Ltd., St. Louis, MO, USA) 500 μL) and stirring was continued for 10 min at room temperature. To the solution was added 28% $NH_4OH$ (85 μL) and the solution was sonicated (BRANSON 3510J-DTH (Scientific Support, Inc., Hayward, CA, USA), 42 kHz, 130 W) for 20 min. After the sonication, tetraethyl orthosilicate (120 μL) was added and the solution was further stirred for 48 h. The colloidal solution was centrifuged for 15 min at 14,000 rpm and the resulting precipitate was washed twice with ethanol and water with each colloidal solution centrifuged for 15 min at 14,000 rpm. The precipitate was dried under vacuum to give LNP(Er)Si (90 mg).

*SI.2. Preparation of LNP(Er)AP*

To a stirred solution of LNP(Er)Si (60 mg) dispersed in ethanol (10 mL) was added 28% $NH_4OH$ (85 μL) and stirring was continued for 10 min at room temperature. To the solution was added 3-aminopropyltriethoxysilane (120 μL) and the solution was stirred for 48 h at room temperature. The colloidal solution was centrifuged for 15 min at 14,000 rpm and the resulting precipitate was washed twice with ethanol and water with each colloidal solution centrifuged for 15 min at 14,000 rpm. The precipitate was dried under vacuum to give LNP(Er)AP (42 mg).

Quantification of the incorporated amino groups was performed by Zhou's method [26]. To a stirred solution of LNP(Er)AP (10.1 mg) dispersed in AcONa buffer (pH 5.5, 5 mL) was added a portion (7.5 mL) of the solution of ninhydrin (2.4 g) in n-propanol (30 mL), ethylene glycol (60 mL), and AcONa buffer (pH 5.5, 10 mL). After 0.6% ascorbic acid (0.5 mL) was added, the solution was heated for 15 min at 100 °C. The solution was diluted to 25 mL and centrifuged for 8 min at 14,000 rpm. The absorbance of the supernatant at 570 nm ($A_{570}$) was 0.242. The calibration curve was made with 2-aminoethanol, in which $A_{570} = 0.5005 \times [\text{-NH}_2]$ (μmol) + 0.0334 ($R^2 = 0.9997$). The amino group incorporation on LNP(Er)AP was thus calculated to be 41 μmol/g.

*SI.3. Preparation of LNP(Er)PEG*

To a stirred solution of PEG (300 mg) in $H_2O$ (6 mL) was added $KMnO_4$ (15 mg) and stirring was continued for 12 h at room temperature. The precipitate was collected by filtration and dried to give a crude carboxylated PEG (PEG-COOH).

To a stirred solution of LNP(Er)AP (25 mg) dispersed in $H_2O$ (700 μL) was added DMAP (13.3 mg, 109 μmol), HATU (24.7 mg, 65 μmol), PEG-COOH (150 mg) was added and stirring was continued overnight at room temperature. The colloidal solution was centrifuged for 15 min at 14,000 rpm and the resulting precipitate was washed with water, ethanol, and then $CH_2Cl_2$ with each colloidal solution centrifuged for 15 min at 14,000 rpm. The precipitate was dried under vacuum to give LNP(Er)PEG.

$A_{570}$ was 0.0500, which is less than the effective value for the amino group measurement and thus the most of amino groups in LNP(Er)AP is thought to be reacted with PEG-attaching reagent.

*SI.4. Preparation of LNP(Er)CHI*

To a stirred solution of chitosan (30 mg) in 1% acetic acid (3 mL) was added LNP(Er)AA (15 mg) and the solution was stirred overnight. The colloidal solution was centrifuged for 10 min at 14,000 rpm and the resulting precipitate was washed with 1% acetic acid and water with each colloidal solution centrifuged for 10 min at 14,000 rpm. The precipitate was dried under vacuum to give LNP(Er)CHI (11.5 mg).

Quantification of the incorporated amino groups was performed by the same method described in the preparation of LNP(Er)AP. The average $A_{570}$ in three times measurements was 0.1304 ± 0.0926. The calibration curve was made with chitosan, in which $A_{570}$ = 0.0007 × CHI (μg) + 0.0716 ($R^2$ = 0.9484). The weight of chitosan on LNP(Er)CHI was thus deduced to be 8.4 ± 3.0 mg/g.

*SI.5. Preparation of LNP(Er)PEI*

To a stirred solution of PEI (81 mg) in water (2 mL) was added LNP(Er)AA (20 mg) and the solution was stirred overnight. The colloidal solution was centrifuged for 10 min at 10,000 rpm and the resulting precipitate was washed twice with water with each colloidal solution centrifuged for 10 min at 10,000 rpm. The precipitate was dried under vacuum to give LNP(Er)PEI (19 mg).

The amount of LNP(Er)PEI for the ninhydrin assay was 3 mg. $A_{570}$ was 0.2167 ± 0.02540 (four times measurements). The calibration curve was made with 2-aminoethanol, in which $A_{570}$ = 0.5005 × [-$NH_2$] (μmol) + 0.0334 ($R^2$ = 0.9997). The amino group incorporation on LNP(Er)PEI was thus calculated to be 120 ± 16 μmol/g.

*SI.6. Preparation of LNP(Er)DAB*

To a stirred solution of LNP(Er)AA (10 mg) dispersed in DMF (700 μL) was added DIPEA (13.6 μL), HATU (9.9 mg), DAB (2.6 μL) was added and stirring was continued overnight at room temperature. The colloidal solution was centrifuged for 10 min at 12,500 rpm and the resulting precipitate was washed twice with DMF with each colloidal solution centrifuged for 10 min at 12,500 rpm. The precipitate was dried under vacuum to give LNP(Er)DAB (7.4 mg).

$A_{570}$ was 0.9265 ± 0.07865 (four times measurements). The calibration curve was made with DAB, in which $A_{570}$ = 0.0848 × [-$NH_2$] (μmol) + 0.0732 ($R^2$ = 0.9997). The amino group incorporation on LNP(Er)DAB was thus calculated to be 1.0 ± 0.09 mmol/g.

*SI.7. Preparation of LNP(Er)ALA*

To a stirred solution ALA (66 mg, 0.5 mmol) in saturated $H_2CO_3$ (5 mL) was added a solution of Fmoc chloride (130 mg, 0.5 mmol) in 1,4-dioxane (0.5 mL) and the solution was stirred overnight. After the solution was acidified with 1 M HCl, it was extracted with ethyl acetate. The organic layer was washed with saturated $NH_4Cl$ and dried over $MgSO_4$. After the inorganics were filtered off, the solution was evaporated and chromatographed on silica (Wako C300, $CH_2Cl_2$:methanol = 150:1) to give FmocALA (55.5 mg, 31%).

To a stirred solution of LNP(Er)AA (15 mg) dispersed in DMF (0.5 mL) was added HATU (9.8 mg), DIPEA (13.6 mg), and FmocALA (9.4 mg) and the solution was stirred overnight. The colloidal solution was centrifuged for 10 min at 12,500 rpm and the resulting precipitate was washed twice with DMF with each colloidal solution centrifuged for 10 min at 12,500 rpm. The precipitate was dried under vacuum to give LNP(Er)FmocALA (12.1 mg).

To a solution of 20% morpholin in DMF (10 mL) was added LNP(Er)FmocALA (10.1 mg) and the solution was stirred for 1 h at room temperature. The colloidal solution was centrifuged for 10 min at 12,500 rpm. The supernatant was kept for Fmoc quantification. The precipitate was washed twice with DMF with each colloidal solution centrifuged for 10 min at 12,500 rpm. The precipitate was dried under vacuum to give LNP(Er) ALA (8.8 mg).

The supernatant was evaporated and the residue was dissolved in $CH_2Cl_2$ (1 mL). The Fmoc-morpholin was quantified by the absorbance at 301 nm ($A_{301}$, $\varepsilon_{301}$ = 7800 $M^{-1}\cdot cm^{-1}$). The average of $A_{301}$ in three measurements was 0.5521 ± 0.05727 and thus ALA incorporation was deduced to be 16 ± 1.6 μmol/g.

*SI.8. Preparation of LNP(Er)FOL*

To a stirred solution of DIPEA (26 μL), HATU (19 mg), folic acid (22 mg) in DMSO (500 μL) was added LNP(Er)DAB (10 mg) and stirring was continued overnight at room temperature. The colloidal solution was centrifuged for 10 min at 12,500 rpm and the resulting precipitate was washed twice with DMSO with each colloidal solution centrifuged for 10 min at 12,500 rpm. The precipitate was dried under vacuum to give LNP(Er)FOL (8.8 mg).

The incorporation of folic acid was quantified by the reported method [27]. Various concentrations of folic acid in 2.5 M acetic acid buffer (pH 4.0, 10 mL), to which 4% $KMnO_4$ (0.05 mL) and 3% $H_2O_2$ (0.1 mL) were added with the interval of 5 min, were subject to the fluorescence measurements ($\lambda_{ex}$ = 365 nm, $\lambda_{em}$ = 446 nm). The fluorescence intensity gave a calibration curve: $F_{446}$ = 6.3594 × [folic acid] (μg) + 4.5114 ($R^2$ = 0.9954). To a stirred solution of LNP(Er)FOL (4 mg) in 2.5 M acetic acid buffer (pH 4.0, 10 mL) was added 4% $KMnO_4$ (0.05 mL) and the solution was stirred for 5 min. To the solution was added 3% $H_2O_2$ (0.1 mL) and the solution was further stirred for 3 min. The colloidal solution was centrifuged for 5 min at 14,000 rpm and the supernatant was diluted

by 100-fold and its $F_{446}$ was 43.246 ± 4.2481 (three times), which deduced the folic acid incorporation of 1.4 ± 0.15 mmol/g.

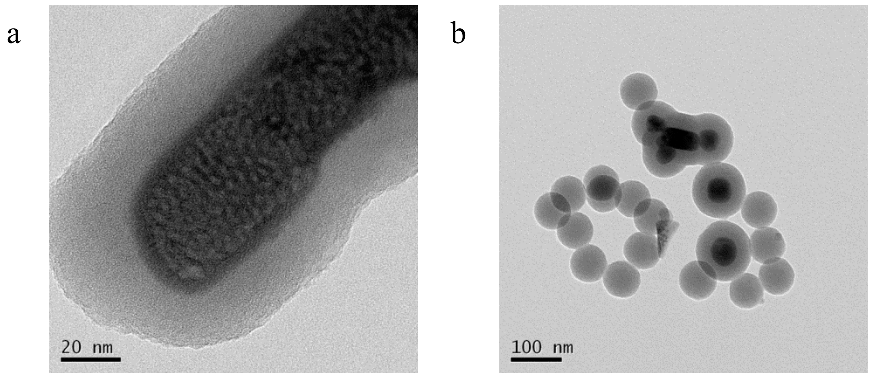


**Figure S1.** Expanded TEM images of (**a**) LNP(Er)AP and (**b**) LNP(Tm)AP.

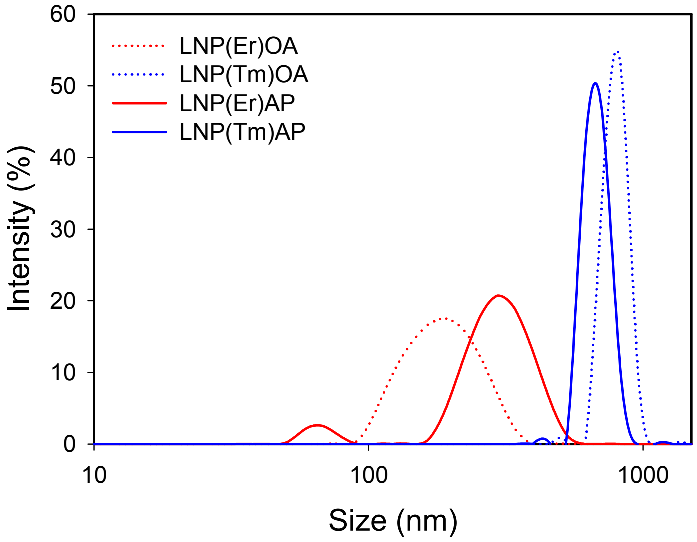


**Figure S2.** DLS of LNP(Er)OA, LNP(Tm)OA, LNP(Er)AP, and LNP(Tm)AP.

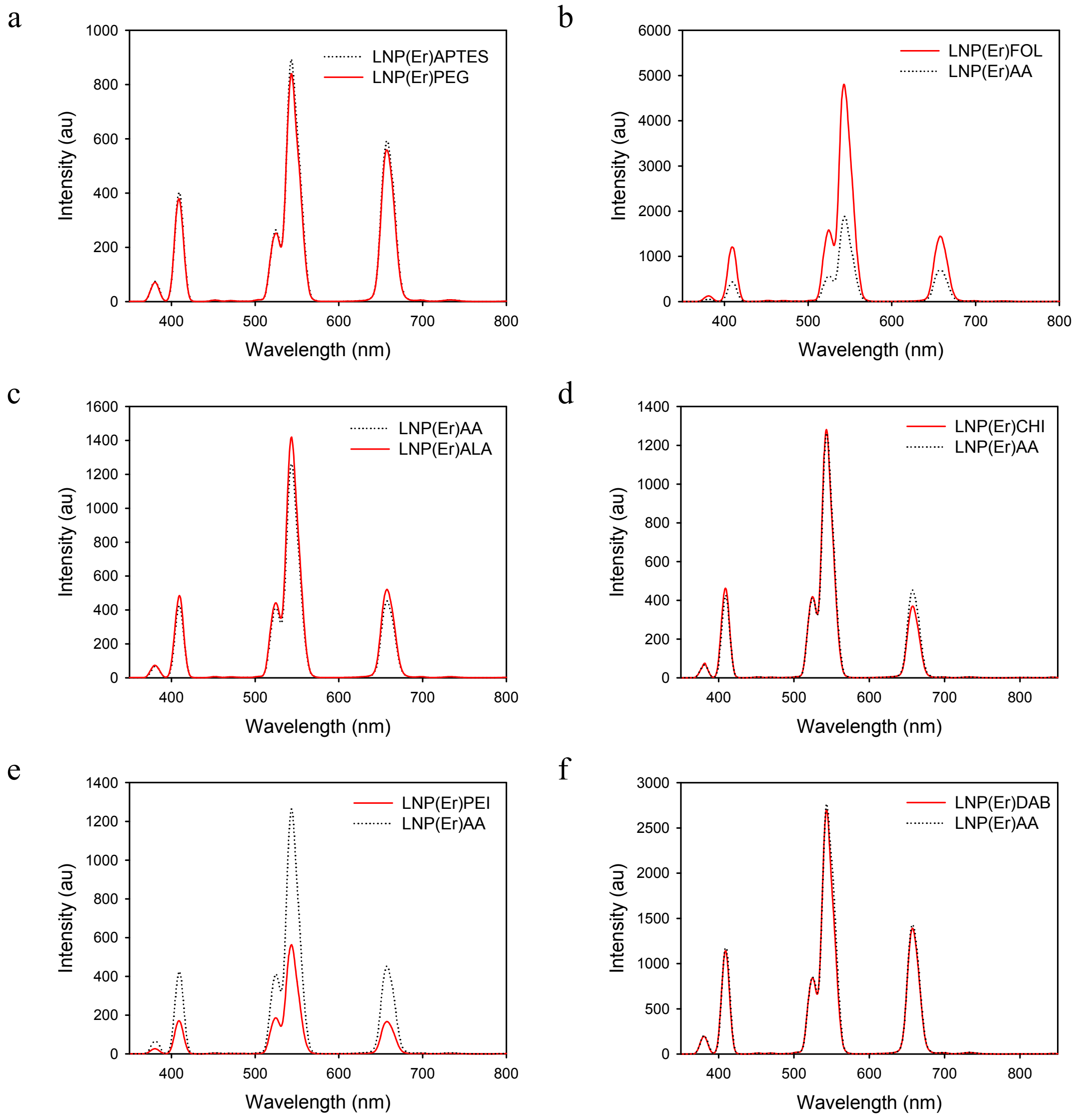


**Figure S3.** Fluorescence spectra ($\lambda_{ex}$ 980 nm) of (**a**) LNP(Er)AP and LNP(Er)PEG; (**b**) LNP(Er)FOL and LNP(Er)AA; (**c**) LNP(Er)ALA and LNP(Er)AA; (**d**) LNP(Er)CHI and LNP(Er)AA; (**e**) LNP(Er)PEI and LNP(Er)AA; and (**f**) LNP(Er)DAB and LNP(Er)AA.

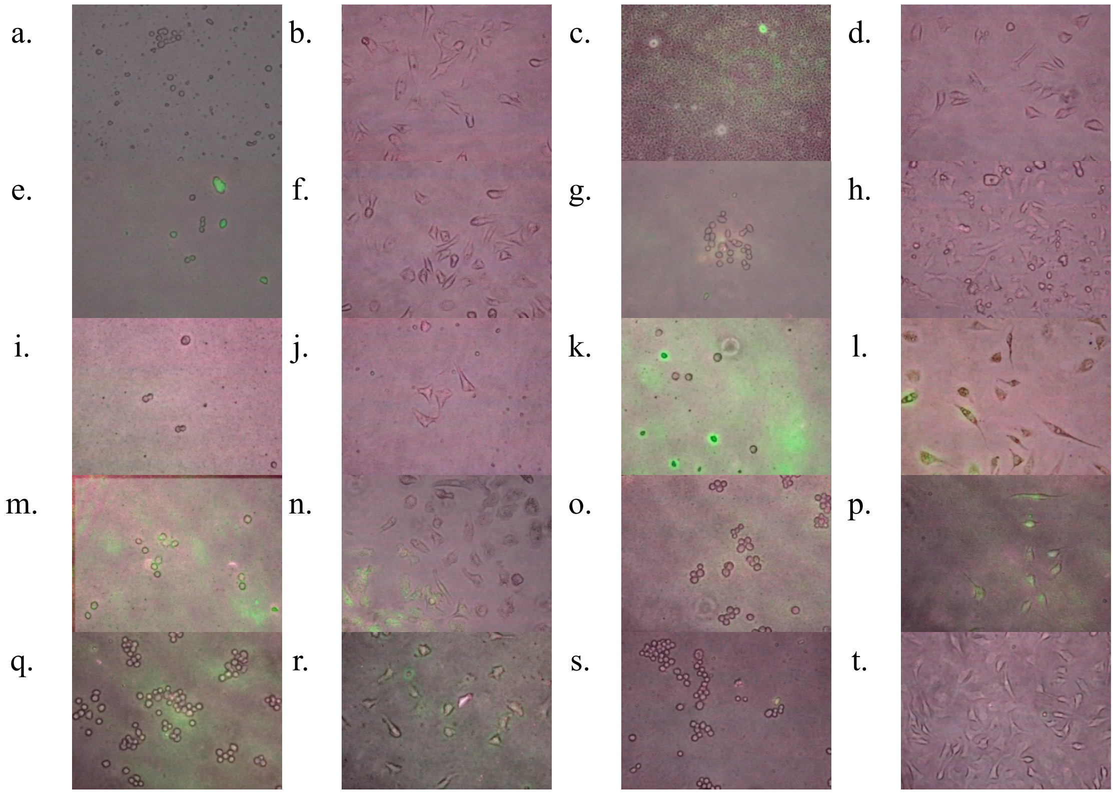


**Figure S4.** The images of the cancer cells incubated with LNPs illuminated with 980 nm laser diode in dark superposed by those under white light: (**a**) MKN45 with LNP(Er)OA; (**b**) HeLa with LNP(Er)OA; (**c**) MKN45 with LNP(Er)Si; (**d**) HeLa with LNP(Er)Si; (**e**) MKN45 with LNP(Er)AP; (**f**) HeLa with LNP(Er)AP; (**g**) MKN45 with LNP(Er)PEG; (**h**) HeLa with LNP(Er)PEG; (**i**) MKN45 with LNP(Er)AA; (**j**) HeLa with LNP(Er)AA; (**k**) MKN45 with LNP(Er)CHI; (**l**) HeLa with LNP(Er)CHI; (**m**) MKN45 with LNP(Er)PEI; (**n**) HeLa with LNP(Er)PEI; (**o**) MKN45 with LNP(Er)DAB; (**p**) HeLa with LNP(Er)DAB; (**q**) MKN45 with LNP(Er)FOL; (**r**) HeLa with LNP(Er)FOL; (**s**) MKN45 with LNP(Er)ALA; (**t**) HeLa with LNPs(Er)ALA.
